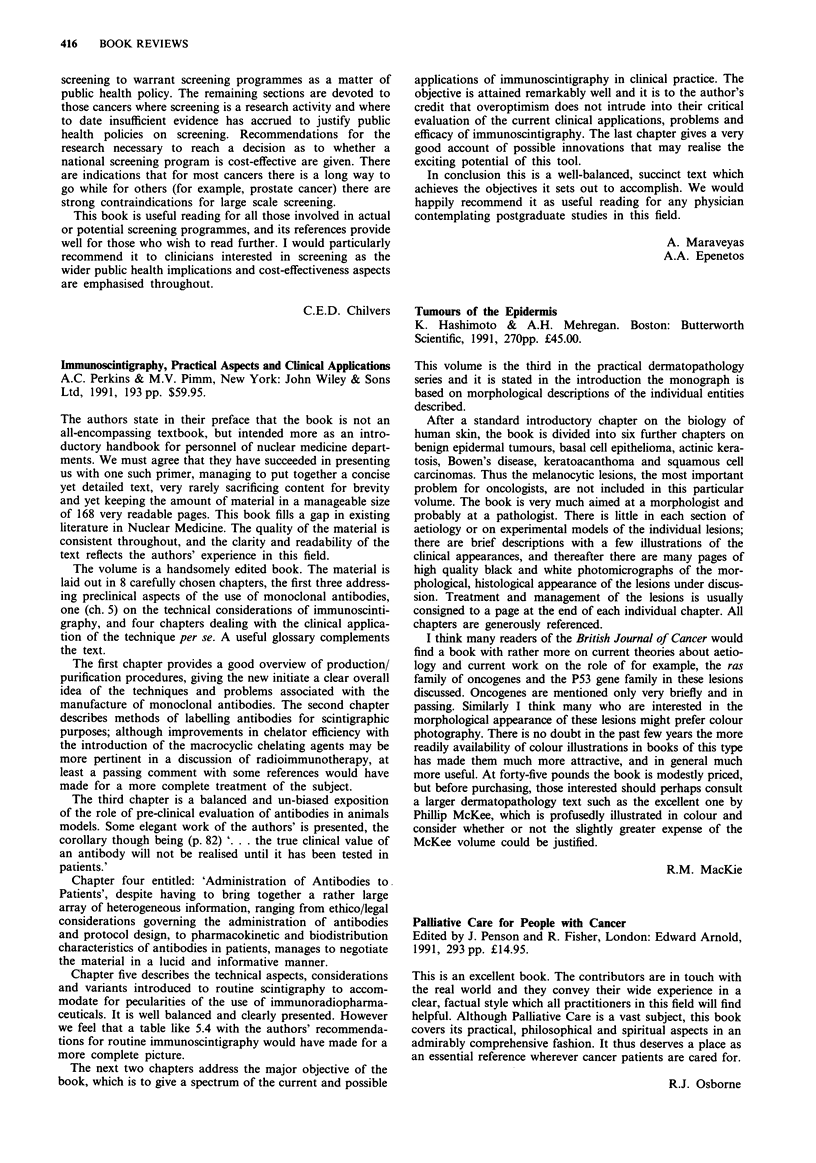# Palliative Care for People with Cancer

**Published:** 1992-08

**Authors:** R.J. Osborne


**Palliative Care for People with Cancer**
Edited by J. Penson and R. Fisher, London: Edward Arnold, 1991, 293 pp. £14.95.

This is an excellent book. The contributors are in touch with the real world and they convey their wide experience in a clear, factual style which all practitioners in this field will find helpful. Although Palliative Care is a vast subject, this book covers its practical, philosophical and spiritual aspects in an admirably comprehensive fashion. It thus deserves a place as an essential reference wherever cancer patients are cared for.

R.J. Osborne